# ASSESSING INNOVATIVE ASSISTIVE TECHNOLOGIES FOR OLDER ADULTS; A KNOWLEDGE AND TECH DEVELOPER'S PERSPECTIVES

**DOI:** 10.1093/geroni/igac059.568

**Published:** 2022-12-20

**Authors:** Robin Syme

**Affiliations:** University of Victoria, Victoria, British Columbia, Canada

## Abstract

**Objective:**

Can Assist is a University of Victoria organization that has been developing assistive technologies (ATs) for almost two decades aimed at developing client-centred broad-impact solutions that address unmet need and help people improve their independence and quality of life. CanAssist’s interest and involvement in this study is predicated on our belief that their approach to technology development align with the criteria needed for determining better tools for evaluating assistive technologies need to be developed and implemented. This is critical to our goal of providing successful customized technology solutions to sustain our clients’ independence and autonomy.

**Methods:**

From the beginning of the project, as a Research User Co-Lead, CanAssist has actively participated in regular advisory committee and expert panel meetings along with several other research activities to co-create all dimensions of the study.

**Results:**

The results from the Rapid Realist Review and preliminary analyses of the interview data with older adults and caregivers have validated the need for more appropriate assessment/evaluation tools to address the varied AT needs of older adults and their caregivers. In particular, the study has provided opportunities for our staff and clients to examine and discuss important factors/processes for successful AT development and implementation.

**Conclusions:**

As a key partner on this implementation science team, CanAssist will use the study’s findings to provide information to our development and management teams on how to appropriately scale-up, spread, and sustain the use of ATs in the health and social care system.

